# Group-based, autonomous, individualized training and testing of long-tailed macaques (*Macaca fascicularis*) in their home enclosure to a visuo-acoustic discrimination task

**DOI:** 10.3389/fpsyg.2022.1047242

**Published:** 2022-11-29

**Authors:** Jorge Cabrera-Moreno, Lena Jeanson, Marcus Jeschke, Antonino Calapai

**Affiliations:** ^1^Cognitive Hearing in Primates (CHiP) Group, Auditory Neuroscience and Optogenetics Laboratory, German Primate Center, Leibniz-Institute for Primate Research, Göttingen, Germany; ^2^Göttingen Graduate School for Neurosciences, Biophysics and Molecular Biosciences, University of Göttingen, Göttingen, Germany; ^3^Auditory Neuroscience and Optogenetics Laboratory, German Primate CenterLeibniz-Institute for Primate Research, Göttingen, Germany; ^4^Institute for Auditory Neuroscience and InnerEarLab, University Medical Center Göttingen, Göttingen, Germany; ^5^Cognitive Neuroscience Laboratory, German Primate Center, Leibniz-Institute for Primate Research, Göttingen, Germany; ^6^Leibniz-ScienceCampus Primate Cognition, Göttingen, Germany

**Keywords:** psychophysics, long-tailed macaque (*Macaca fascicularis*), primate learning, machine learning, home-cage training

## Abstract

In recent years, the utility and efficiency of automated procedures for cognitive assessment in psychology and neuroscience have been demonstrated in non-human primates (NHP). This approach mimics conventional shaping principles of breaking down a final desired behavior into smaller components that can be trained in a staircase manner. When combined with home-cage-based approaches, this could lead to a reduction in human workload, enhancement in data quality, and improvement in animal welfare. However, to our knowledge, there are no reported attempts to develop automated training and testing protocols for long-tailed macaques (*Macaca fascicularis*), a ubiquitous NHP model in neuroscience and pharmaceutical research. In the current work, we present the results from 6 long-tailed macaques that were trained using an automated unsupervised training (AUT) protocol for introducing the animals to the basics of a two-alternative choice (2 AC) task where they had to discriminate a conspecific vocalization from a pure tone relying on images presented on a touchscreen to report their response. We found that animals (1) consistently engaged with the device across several months; (2) interacted in bouts of high engagement; (3) alternated peacefully to interact with the device; and (4) smoothly ascended from step to step in the visually guided section of the procedure, in line with previous results from other NHPs. However, we also found (5) that animals’ performance remained at chance level as soon as the acoustically guided steps were reached; and (6) that the engagement level decreased significantly with decreasing performance during the transition from visual to acoustic-guided sections. We conclude that with an autonomous approach, it is possible to train long-tailed macaques in their social group using computer vision techniques and without dietary restriction to solve a visually guided discrimination task but not an acoustically guided task. We provide suggestions on what future attempts could take into consideration to instruct acoustically guided discrimination tasks successfully.

## Introduction

Training non-human primates (NHP) in various husbandry and veterinary procedures is essential to animal behavior management in most captive settings. Positive reinforcement training (PRT) (Skinner, 1938) is the most efficient and ethical technique to train a wide variety of behaviors as it rewards the animals for desired behaviors while ignoring unwanted ones (Westlund, 2015). The standard procedure in PRT training is to break down a desired final behavior into small pieces that can be gradually and sequentially taught to the animal. However, training behaviors required to perform typical experimental tasks in sensory-motor systems research and cognitive neuroscience represent a more significant challenge for classical PRT training. First, most PRT protocols need human trainers to start and end each session and, in some cases, each trial (manual shaping). Besides the time cost—namely that a human trainer can only handle a single animal at a time—there is an unavoidable diversity of training strategies that trainers apply for different animals, ultimately making comparisons across animals and replicability of results challenging (Berger et al., 2018). Finally, in neuroscientific laboratories, NHPs are usually taken from the home cages to insulated experimental setups where they are trained in isolation, potentially reducing the training time and the natural species-specific behavioral repertoire that an animal can express.

Therefore, we would like to argue that the optimization of training protocols has the potential to enhance animal welfare while increasing the standardization of training and ultimately broadening the scope of scientific research. Toward such aims, several studies have already reported various optimization of behavioral training (Calapai et al., 2017, 2022; Berger et al., 2018; Butler and Kennerley, 2019; Walker et al., 2019; Sacchetti et al., 2021) across two important NHP models used in neuroscience, rhesus macaques (*Macaca mulatta*), and common marmosets (*Callithrix jacchus*). However, to the best of our knowledge, there is a lack of reported attempts to develop automated training and testing protocols for long-tailed macaques (*Macaca fascicularis*), a ubiquitous NHP model in neuroscience and—in particular—pharmaceutical research. Long-tailed macaques are 38–55 cm large cercopithecine primates native to Southeast Asia. Animals of this species live in complex social groups—multi-male/multi-female, 6 to 40 individuals—with a dominance hierarchy among females that can be passed through generations of matrilines (Van Noordwijk and Van Schaik, 1985; van Noordwijk and van Schaik, 1999). Due to their close physiological proximity to humans, long-tailed macaques represent a valuable model for biomedical research, especially for basic research studies in disease pathology and treatment, vaccine development, immunology, and neuroscience. Hence, the refinement of protocols to evaluate cognition and behaviors in long-tailed macaques is highly important for phenotyping in treatment development and understanding cognition, affection, and social processes.

In this study, we describe a computerized, automated protocol for training and testing captive long-tailed macaques in their social group. Our approach achieves self-paced, step-wise, individualized training employing picture-based animal identification at the beginning of each trial, which is instrumental in adjusting the training based on the animals’ trial-by-trial proficiency. With this approach, no human interaction with the animals is needed, and only minimal maintenance and supervision are required, with presumed positive repercussions on the data quality and the results’ replicability. Furthermore, we also argue that removing physical constraints while also keeping the animals in their housing environment with their social group opens the possibility of investigating a broader range of more complex behaviors, including social interactions. Home-cage training also enables the opportunity to record neural activity for extended periods by using wireless recording technologies (Chestek et al., 2009 ; Borton et al., 2013; Zhou et al., 2019 ).

Here, we report the results from 6 long-tailed macaques navigating an Automated Unsupervised Training (AUT) procedure to reach a visuo-acoustic two-alternative choice (2 AC) task. We show that our animals can successfully navigate an AUT procedure to learn a visually guided 2 AC on a touchscreen but fail to do the same based on acoustic information.

## Results

**Figure 1:**
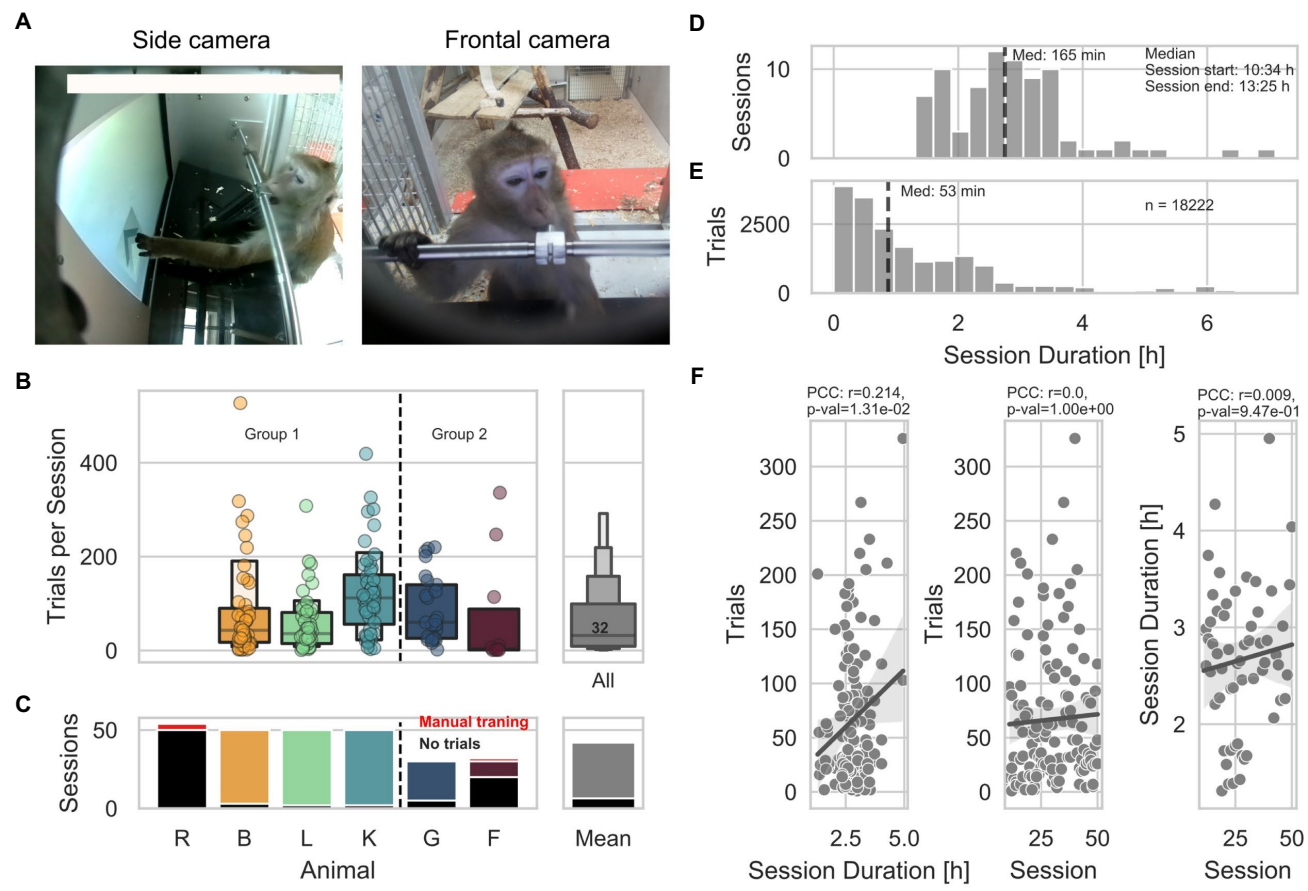
General engagement across sessions. **(A)** Pictures of animal L interacting with the LXBI device. The left picture shows the view from the side camera used for surveillance during sessions. The right picture shows the view from the frontal camera used for animal identification. **(B)** Left panel shows the number of trials per session across animals. The right panel shows the distribution across all animals, with a median of 32 trials per session (IQR = 90 trials). **(C)** Left panel shows the number of sessions across animals. Red indicates the amount of manual training sessions conducted in separation from the rest of the group. Black indicates the amount of sessions with no trials. The right panel shows the mean across animals. **(D)** Distribution of all session durations. The dashed line indicates the median of the distribution. **(E)** Distribution of trial initiation across session duration. The dashed line indicates the median of the distribution. **(F)** From right to left. Distribution of number of trials per animal as a function of session duration, shows a significant positive correlation (partial Pearson’s correlation, *n* = 135, *r* = 0.213, CI95% = [0.05, 0.37], *p* = 0.01). Distribution of number of trials per animal as a function of session number, shows non-significant correlation (partial Pearson’s correlation, *n* = 135, *r* = 0.00008, CI95% = [−0.17, 0.17], *p* = 0.99). Distribution of session duration as a function of session number shows no significant correlation (partial Pearson’s correlation, *n* = 59, *r* = 0.0088, CI95% = [−0.25, 0.27], *p* = 0.94).

In this study, 6 female long-tailed macaques (*Macaca fascicularis*) housed in two groups, see Table 1, were given access to a touchscreen device attached to their home cage. At the same time, solid food and fluid were provided *ad libitum*. All animals had previous exposure to a similar device during a separate experiment a year prior to this study and were already familiar with the basics of touchscreen interaction. Sessions were mainly autonomously conducted with sporadic human supervision (except for animals R and F trained by an experimenter in 4 and 2 shaping sessions, respectively; see below). Upon the initiation of each trial, throughout the autonomous and unsupervised training procedure, a machine learning algorithm identified the animals from a picture taken by a camera placed on top of the screen (Figure 1A). In this way, animals could progress in step-wise training between and within sessions [see methods: Automated unsupervised training (AUT)].

**Table 1:** Metadata and summary statistics.

Group	Name	Sex	Age [years]	Sessions [total]	Individual sessions	Trials [total]	Sessions with 0 trials
1	Renate	f	22	54	4	0	54
1	Bella	f	20	50	0	1,672	3
1	Leni	f	21	50	0	1,438	2
1	Kuemmel	f	10	50	0	4,303	2
2	Granny	f	10	30	0	1,596	5
2	Fenja	f	17	32	2	264	20

### General engagement across sessions

Animals’ engagement varied within and between sessions, with a median of 32 trials (IQR = Q3-Q1 = 90) per session across 50 and 30 sessions per Group 1 and Group 2, respectively (Figure 1B). Animals R and F underwent individualized shaping sessions to improve touching accuracy (for 4 and 2 sessions, respectively). The total number of sessions is the number of times the device was offered to the group, regardless of the number of interactions. Except for animal R, which did not perform a single trial across all the sessions, the mean number of sessions with 0 trials per animal is 6 (Figure 1C). The session duration ranged from 1.3 to 7 h with a median of 2 h and 45 min (starting and ending at 10:34 h and 13:25 h, respectively − Figure 1D). To describe potential habituation effects, we statistically evaluated whether the number of trials per animal varied as a function of session duration or session number and whether the number of trials per hour varied across consecutive sessions. Initial sessions during which solely pictures (see methods) were taken were excluded from this analysis as they were designed to be longer in duration and easier to solve by the animals. We found a significant positive correlation between the number of trials each animal performed and the session duration (partial Pearson’s correlation, *n* = 135, *r* = 0.213, CI95% = 0.05, 0.37, *p* = 0.01; Figure 1F), suggesting that longer sessions lead to more trials. On the other hand, we found no significant correlation between the number of trials performed and the session number (partial Pearson’s correlation, *n* = 135, *r* = 0.00008, CI95% = −0.17, 0.17, *p* = 0.99); as well as between the session duration and the sessions number (partial Pearson’s correlation, *n* = 59, *r* = 0.0088, CI95% = −0.25, 0.27, *p* = 0.94), suggesting that animals did not lose interest in the experiment across consecutive sessions while access to the device remained consistent. Finally, by looking at the distribution of trials across all sessions and all animals, we found that animals mostly engaged during the first 2 h of the sessions, performing 50% of the trials within the first 53 min (Figure 1E).

### Visuo-acoustic automated unsupervised training

In this study, we adapted a visuo-acoustic automated unsupervised training protocol (AUT) we previously used to train marmoset monkeys (Calapai et al., 2022). Here, 5 long-tailed macaques belonging to 2 groups underwent an AUT comprised 49 training steps. The AUT protocol was designed to (1) improve touch precision (milestone *size*), (2) spatial touch precision and tolerance to acoustic stimuli (milestone *location-sound*), and (3) train a 2 alternative audio-visual association (milestone *distractor*). Training data for animal R are not available as the animal never interacted with the device.

**Figure 2:**
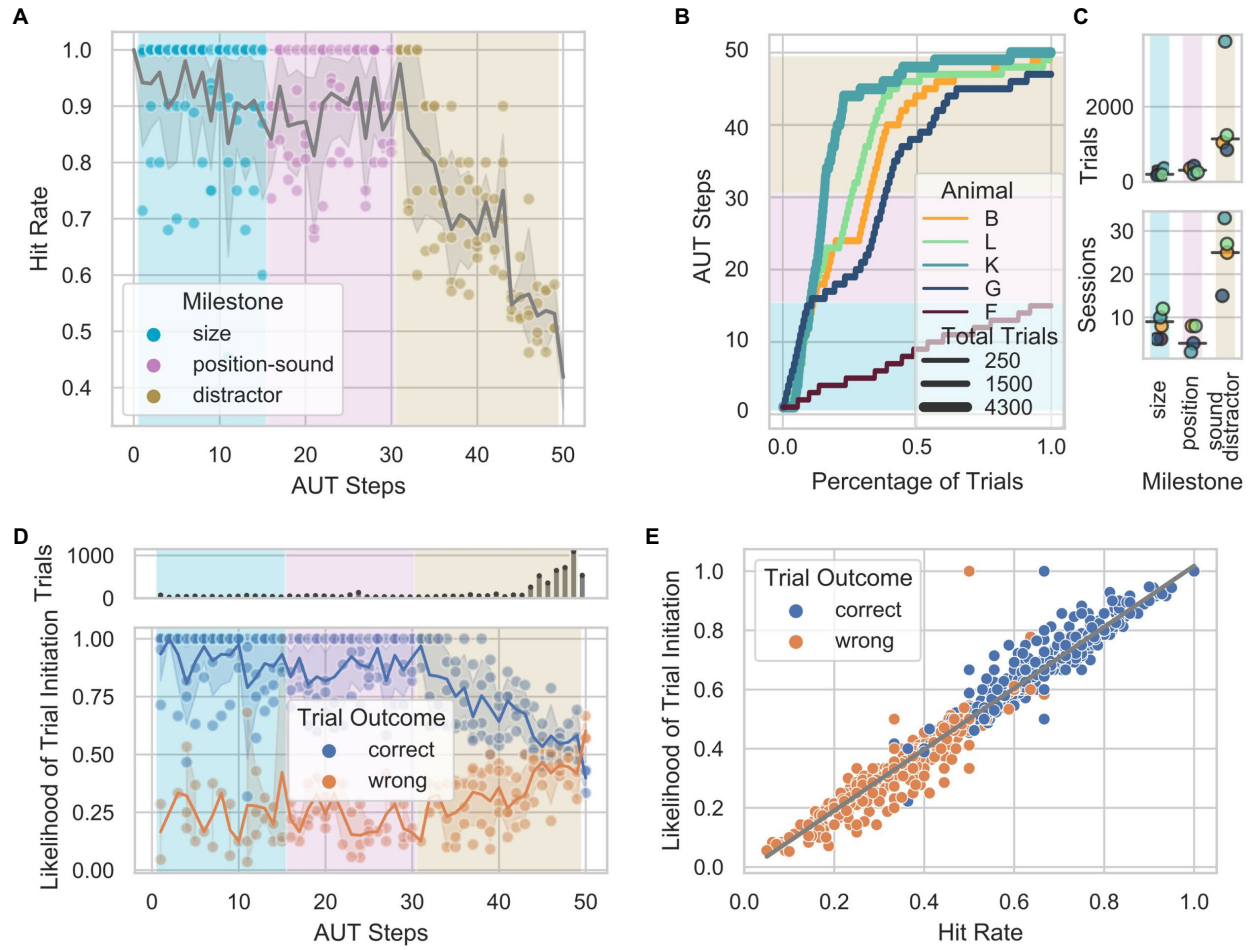
Performance through the Automated Unsupervised Training (AUT) protocol. **(A)** Hit rate as a function of AUT steps per animal. Gray shade represents 95% confidence interval of the mean across animals. **(B)** Animal progress through the steps of the AUT protocol. Background colors indicate the milestones. **(C)** From top to bottom, number of trials and number of sessions as a function of milestones across animals. **(D)** Distribution of the likelihood of trial initiation as a function of hit rate in blocks of 100 randomly selected trials across animals. The upper panel shows the number of trials per step. **(E)** Highly significant positive correlation between the hit rate and the likelihood of initiating a trial when controlling for the non-uniform number of trials across steps (partial Pearson’s correlation, *n* = 840, *r* = 0.98, CI95% = [−0.98, 0.98], *p* =5e-18).

An algorithm that monitored the animals’ hit rate within a sliding window of 10 trials loaded the subsequent step when 8 out of 10 trials were correct or the previous step when 2 out of 10 trials were correct, modulating the task difficulty as a function of the animal’s performance. Although the design of the AUT aimed to individualize and smoothen the transition between steps according to the animals’ learning progress, certain milestones required more trials to be acquired. Therefore, different hit rates can be observed across AUT steps and milestones (Figure 2A). An important feature to note is the consistent decrease in performance starting with the last milestone, during which a visual distractor was added. Except for animal F, which did not overcome the milestone size (with 250 trials and 54 sessions), 4 out of 5 animals reached the *distractor* milestone (B, L, K, G) and successfully acquired the visual part of the last milestone. In contrast, none successfully acquired the acoustic part. To visualize the learning progress through the milestones of the AUT across animals with potentially different engagement levels, we quantified the number of trials as a function of the total trials performed (Figure 2B). The animals needed an average of 200, 304, and 1,141 trials; and 9, 4, and 25 sessions to overcome the *size*, *location-sound*, and *distractor* milestones, respectively (Figure 2C). This suggested that two of these milestones (*size* and *position-sound*) were easier to solve than the final milestone (*distractor*), which might have needed a smoother training set of steps than the one used in the current study. To assess whether individual animals’ performance influenced subsequent task engagement, we analyzed the likelihood of initiating a trial after a correct or wrong response within the first 30 s following a response. We found that the likelihood of starting a trial after a correct response remained stable during the first two milestones (*size* and *location-sound*). In contrast, it consistently changed in the milestone *distractor*, decreasing from 90 to 55%. This pattern is mirrored by the likelihood of initiating another trial after a wrong trial, going from 25% in the initial milestones to 40% in the distractor (Figure 2D). The same was observed when controlling for the non-uniform number of trials across steps by recomputing the likelihood based on an equal number but randomly selected sample of trials belonging to all steps (see methods). We found a significant positive correlation between the hit rate and the likelihood of initiating a trial (Figure 2E), suggesting that the animals’ engagement is heavily dependent on short-term performance as lower hit rates over time tend to promote similar trial initiation for correct and wrong trials.

## Two-alternative visuo-acoustic discrimination

**Figure 3:**
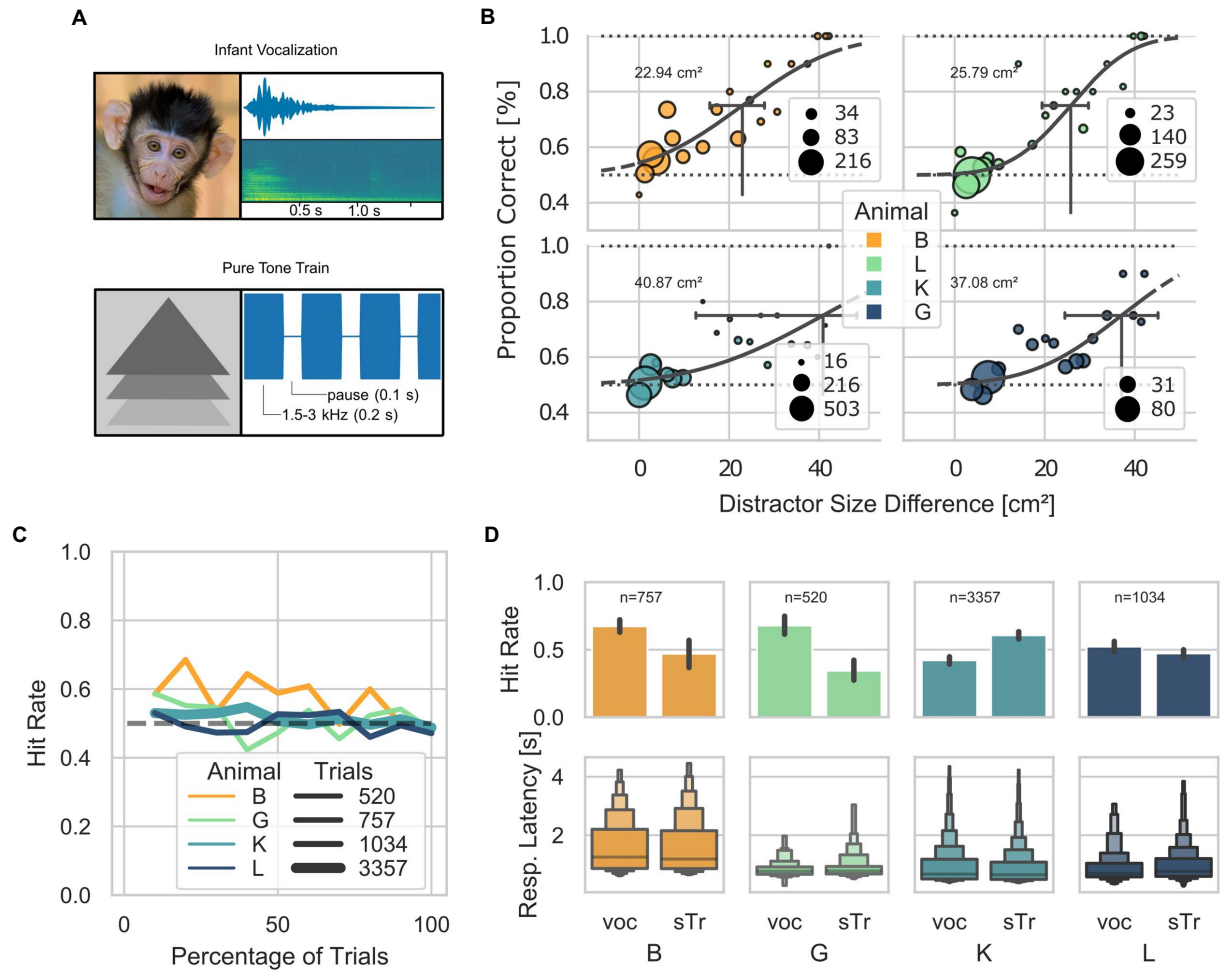
Visually guided discrimination task. **(A)** Visual and acoustic stimuli used across the AUT (milestones *position-sound* and *distractor*) and in step 50. Visual stimuli are shown on the left, and the spectro-temporal information of the acoustic stimuli is shown on the right. The pure tone train was 4 kHz. **(B)** Psychometric curves for the minimum size difference between distractor and target, calculated as the proportions of correct trials across steps of the AUT. 95% confidence intervals (CI) are indicated with black horizontal lines (Animal B: threshold 22.94 cm^2^; CI between 15.7 and 26.7; L: 25.79 cm^2^, CI between 19.4 and 28.9; K: 40.87 cm^2^, CI between 12.6 and 46.4; G: 37.08 cm^2^, CI between 24.4 and 43.3). **(C)** Hit rate as a function of the percentage of trials performed by each animal (after step 44 where all animals mostly performed below 60% hit rate), grouped into bins of 10%. The thickness of the lines represents the number of trials. The dashed line at 0.5 represents the chance level. **(D)** Letter-value plots show the reaction times for each stimulus across animals after step 44. The central box represents the 1st quartile, 2nd quartile and 3rd quartile. No statistical difference was found between the response latencies between stimuli at a Bonferroni *post-hoc*-corrected Kruskal–Wallis Test (B *p* = 0.19, G *p* = 0.17, K *p* = 0.18, L *p* = 0.09).

From steps 31–49, the AUT protocol attempted to train the animals to discriminate between a target and distractor simultaneously presented on the screen (Figure 3A) based on two cues: a visual cue, the difference in the size of the visual stimuli; and an acoustic cue, the specific sound played throughout the trial. Animals could use either cue to determine the target of a given trial. However, from step 50 onward, only the acoustic cue was present as the target and distractor had the same size. While 4 out of 5 animals reached step 50, none had a performance above chance at this stage of the training. This suggests that animals did not use the acoustic cue to identify the target of a given trial but relied exclusively on the stimuli’s size difference. A psychometric estimation based on the proportion of correct trials across steps 31 to 50 revealed that the minimum detectable size differences between the target and distractor are: 22.94 cm^2^, 25.79 cm^2^, 40.87 cm^2^, and 37.08 cm^2^ for animals B, L, K, and G, respectively (Figure 3B). In addition, animals showed a stable hit rate (around the chance level) once the difference between the target and the distractor was around 0.8 cm^2^ (step 44; Figure 3C). After step 44, animals B, and G, showed a bias for the vocalization and animal K for the simple train (Figure 3D). Also, no significant difference in the response latency between the two stimuli was found (Kruskal–Wallis, Bonferroni corrected B *p* = 0.19, G *p* = 0.17, K *p* = 0.18, L *p* = 0.09.

### Face identification performance

**Figure 4:**
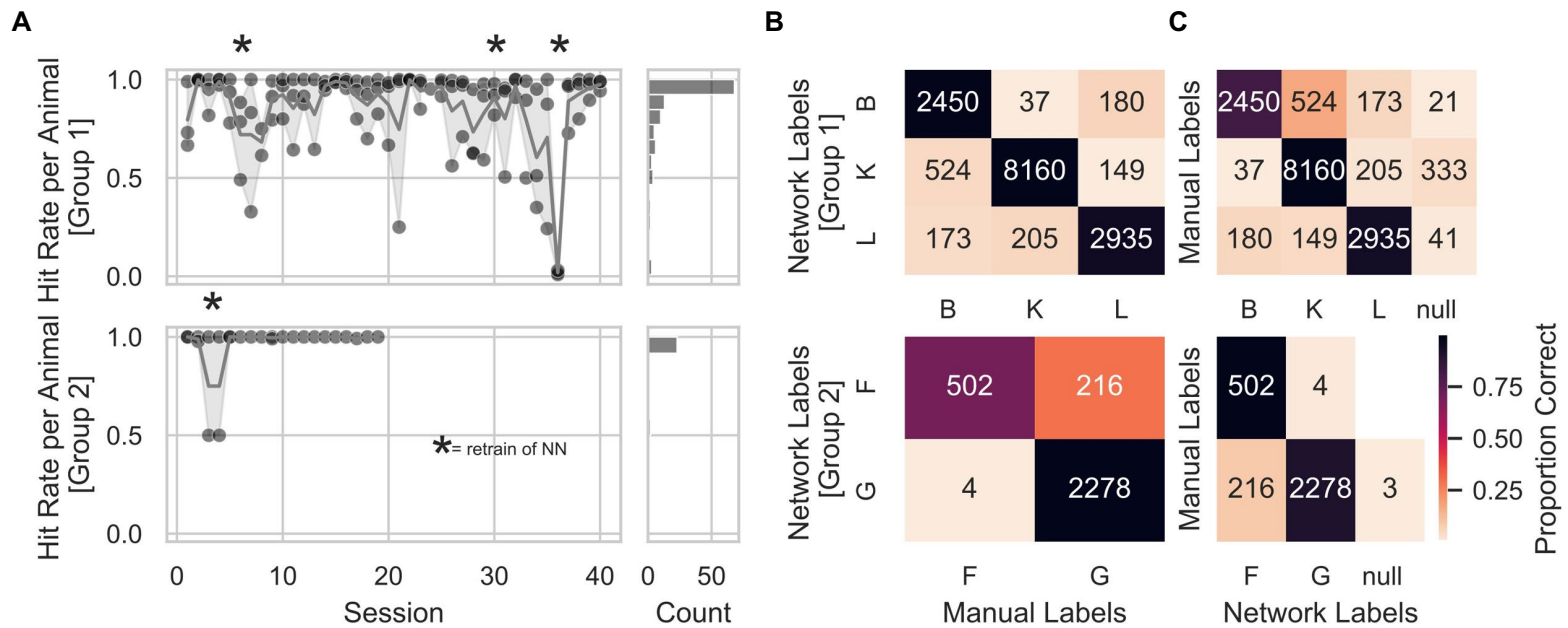
Animal identification accuracy. **(A)** Animal identification accuracy across sessions for Group 1 on the upper right panel and Group 2 on the lower right panel. The right panels show a count histogram for both groups. **(B)** Individual animal identification accuracies were calculated as the number of times labels from the network-matched manual labels, divided by the total number of network labels for that animal. Accuracies for Group 1 were around 90%. At the same time, for Group 2, animal G held an identification accuracy of almost 100% and animal F of 70%. **(C)** General measure of accuracy for each animal, calculated by dividing the number of times labels from the network-matched manual labels by the total amount of manual labels for that given animal. Again, accuracies were above 90% in Group 1 and between 77 and 89% in Group 2. An additional animal label, called *null*, was assigned to those pictures where the animal’s identity was unclear (animals triggering a trial by accident, e.g., with their back). Numbers inside the heatmap represent the number of trials from which the hit rate was calculated.

In order to individualize the automatized training protocol for each animal, we trained a convolutional neural network with a structure optimized for object categorization (LeCun et al., 2015), to identify the animals at the start of each trial. We manually labeled all pictures offline to assess the neuronal network’s animal identification performance. We observed stable animal identification performance of the network across consecutive sessions for both groups (Figure 4A). The network was retrained after sessions: 5, 29, and 35 for Group 1, and after session 4 for Group 2 (indicated with stars in Figure 4A) to expand the training set and potentially prevent drops in identification accuracy. Session 36 of Group 1 was removed from the analysis due to a technical problem with the training of the network (the animals’ labels were swapped inadvertently). Figure 4B shows that individual animal identification accuracies for Group 1 were around 90%, while for Group 2, animal G held an identification accuracy of almost 100% and animal F of 70%. The accuracy for a given animal was calculated as the number of times labels from the network-matched manual labels divided by the total number of network labels for that animal. Furthermore, we computed a more general measure of accuracy for each animal by dividing the number of times labels from the network-matched manual labels by the total amount of manual labels for that given animal. We found this general accuracy above 90% in Group 1 and between 77 and 89% in Group 2 (Figure 4C). Finally, to avoid that wrongly identified animal’s influenced a given animals’ progress within the AUT, we took and fed to the network a second picture at the end of each trial before computing the AUT progression. This allowed online identification of trials with different labels from start and end to prevent potential problems with the AUT progression. In addition, this prevented 319 wrong assignments of the trial outcome out of a total of 8,784 trials.

### Animal turn-taking

**Figure 5:**
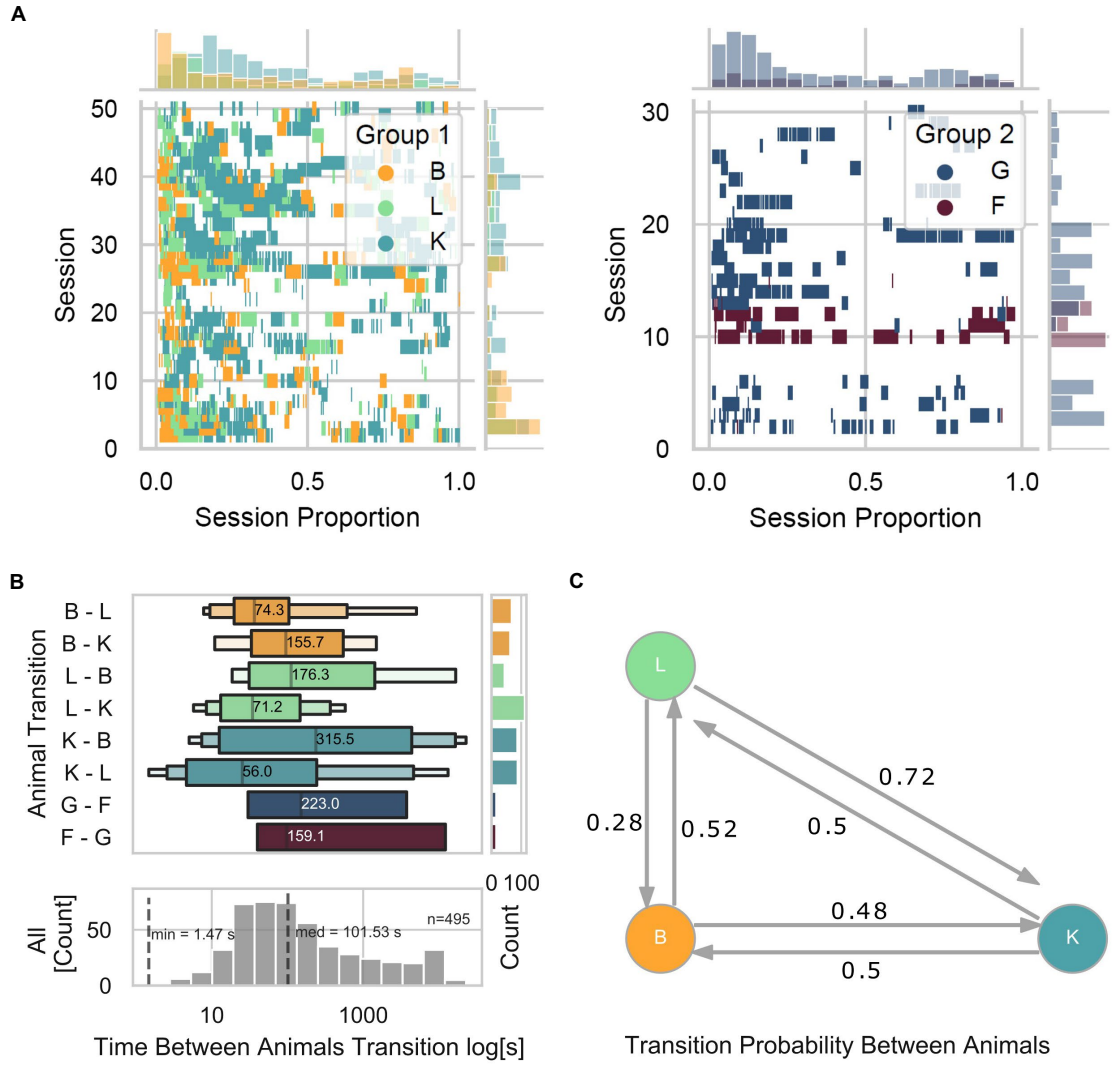
Turn-taking. **(A)** Event plot showing each animal’s individual trial initiation as a function of session proportion. Left panel for Group 1 and right panel for Group 2. Marginal plots show the density histograms of trial initiation instances across sessions on the ordinate and within sessions on the abscissa. **(B)** The upper panel shows the time distribution between animal transitions across all animals. The lower panel shows the distribution when merging all animals. The marginal plot is a count histogram for the number of transitions. **(C)** Markov transition matrix, showing the probability of transitions among animals.

The online animal identification algorithm, allowing for individualized training and testing of our animals living in social groups, allowed for assessment of the animal-device interaction from a group-level perspective. First, we observed that the level of engagement with the device (taken as the number of interactions as a function of time within a session) is consistently higher at the beginning and lower toward the end (Figure 5A). Specifically, in Group 1, we found that within each session, animal B was often the first to interact with the device, followed by animal L, and later by animal K. Moreover, within and across sessions, we observed 463 total transitions from a given animal to a different animal, with a median interval of 101.53 s (Figure 5B). We found that transitions from L to K occurred the most (112), while L to B the least (44), in contrast with other transitions that occurred relatively evenly (B to L and B to K with 63 and 68 transitions, respectively; K to B and K to L with 87 and 88 transitions, respectively). To graphically describe the transition probability among animals, we calculated a Markov transition matrix for Group 1 (Figure 5C) and statistically assessed whether transitions were due to random transitions between animals. Toward this, we quantified the probability of obtaining similar results with shuffled data (1,000 repetitions) while keeping the same amount of interactions as in the original data. Except for transitions of animal K to L (two-sided permutation test; *p* = 0.123) and K to B (two-sided permutation test; p = 0.123), none of the transitions can be explained by chance alone (two-sided permutation test; B to K *p* = 0.055, B to L p = 0.055, L to B *p* = 0.001, and L to K p = 0.001). These results suggest a preferred turn-taking order with which animals interacted with the LXBI. Such structure cannot be explained by chance, and is likely the product of complex social dynamic interactions within the group. It remains unclear whether the LXBI created such structure or whether the structure existed before and the animals used it as it would naturally happen in naturalistic foraging situations.

## Discussion

Non-human primates (NHP) play an essential role in biomedical research due to their physiological, psychological, and cognitive proximity to humans. However, the requirement to manually train NHPs to understand complex rules and perform complex behaviors bears several caveats. Among them are the inter-experimenter variability of training, the difficulty in generalizing the results, the time and personnel needed, as well as ethical considerations related to the animals’ well-being. In an effort to address these issues, we designed a touchscreen-based, autonomous, individualized experimental protocol to train and test long-tailed macaques directly in their home enclosure without fluid/food control or social separation that integrates trial-by-trial animal identification employing a convolutional neural network. Six female long-tailed macaques, across two separate captive groups, underwent daily training sessions (Monday to Friday) for around 3 h on a touchscreen device attached to their home cage. Our results suggest that: (1) captive long-tailed macaques successfully learn a visually guided discrimination task with autonomous protocols, but demonstrated that more sophisticated approaches than the gradual implicit sound-to-stimulus association we employed are needed for acoustically guided discrimination; (2) animals engage with the device without the necessity of food/fluid control, but such engagement strongly correlates with success rate, as interactions decrease as the task becomes more difficult; (3) picture-based animal identification through machine learning was stable across several months and animals, making it a reliable and non-invasive technique for animal tagging to achieve individualized training without social separation; and (4) it is possible to assess group-level dynamics (such as turn-taking) in socially housed non-human primates.

### Visuo-acoustic automated training

Our home-cage, automated training protocol was designed based on similar experimental protocols developed for NHPs across the last two decades (Mandell and Sackett, 2008; Bullock and Myers, 2009; Tulip et al., 2017; Butler and Kennerley, 2019; Walker et al., 2019; Bala et al., 2020; Wither et al., 2020; Sacchetti et al., 2021). Specifically, it shared the structural design of the apparatus described for Rhesus macaques (Berger et al., 2018) while replacing costly hardware (Apple computers) with an open-source system (Raspberry Pi computers), allowing for more straightforward modification and expansion of the system by others. It additionally used a visuo-acoustic protocol developed for common marmosets (Calapai et al., 2022). Furthermore, our protocol relies on computer vision technology for the identification of subjects on a trial basis, which could, in principle, allow for testing subjects in natural settings. Our results suggest that long-tailed macaques can be trained in an automated manner to perform basic visually guided tasks using a touchscreen system but failed to generalize to an acoustically guided 2 AC task. These findings are in line with previous reports that showed that long-tailed macaques could perform a stimulus-directed touch behavior using a touchscreen system engaging consistently over several sessions (Bullock and Myers, 2009; Rice et al., 2017). However, previous reports have shown that macaques (*Macaca fascicularis*, *Macaca fuscata*, and *Macaca nemestrina*) are indeed able to solve acoustic discrimination tasks (Kuhl and Padden, 1983; Petersen et al., 1984; Brosch et al., 2004; Furuyama et al., 2017). However, differences in stimulus type (human vocalizations versus conspecific vocalizations), setup conditions (attenuated sound chamber versus animal colony, lever versus touchscreen), and testing paradigms (Go No-Go versus 2 AC) might account for differences in performance (Waskom et al., 2019), preventing direct comparison across studies. When comparing our results to those reported for common marmosets using a similar system (Calapai et al., 2022) where 9 out of 11 marmosets learned to discriminate conspecific vocalizations from pure tone trains using a 2 AC or 3 AC paradigm, we found a substantial difference in the engagement of the animals when low hit rates are observed. Our analysis showed that even though the number of trials performed per animal remained relatively constant over sessions (engagement), the likelihood of performing more trials in a row depended on the performance. We argue that this change in engagement dynamics might have contributed to the failure to acquire the visuo-acoustic 2 AC from the long-tail macaques because it hindered the necessary exposure time required to learn the discrimination. Regardless of this change, our aim was to elicit an implicit audio-visual association during the later steps of an automated training protocol. Instead, animals ignored or discounted the acoustic information presented and focused exclusively on the visual information (i.e., the difference in stimulus size). Finally, from the necessity to train one long-tailed macaque in the current study and personal communication with the authors that previously exposed the same group of long-tailed macaques to a similar touchscreen device in the context of a different study (Cassidy et al., 2021), we would argue that naïve long-tailed macaques could be trained to interact with our device.

### Level of engagement with automatized training protocols

Five out of six animals interacted consistently with the device across several months, in sessions of 3 h duration during which fluid and food were available *ad libitum*. This was presumably due to the sugary fluid reward delivered by the device in combination with the provision of an activity that provided a form of cognitive enrichment (Murphy et al., 2003; Tarou and Bashaw, 2007; Clark, 2017, 2022; Calapai et al., 2022). On the other hand, we found that engagement strongly depended on short-term performance levels as the likelihood of initiating a trial decreased with an increase in difficulty and throughout the training section in which the task gradually moved from visuo-acoustic to acoustic only. This dependency should be considered for future experiments, especially when generalizations across sensory modalities are needed for experimental purposes. Interestingly, a similar dependence was observed for some individuals at the same stage of the AUT protocol in a previously published marmoset study (Calapai et al., 2022). Finally, while our animals were aged between 10 and 22 years old, considered already “aged” animals (Veenema et al., 1997, 2001), the reported marmosets were significantly younger (2–7 years old), marmosets are often referred to as “aged” at 8 years of age (Abbott et al., 2003). Because the cognitive decline in aging NHPs is well-demonstrated and particularly relevant for translational neuroscientific research (Herndon et al., 1997; Smith, 2004; Nagahara et al., 2010; Gray and Barnes, 2019; Sadoun et al., 2019; Lacreuse et al., 2020), our approach could be helpful to assess and describe aspects of cognitive decline in captive NHPs in a standardized way.

### Animal recognition with machine learning and computer vision

Reliable identification of individuals in socially housed settings and operating device for automated training and cognitive assessment represents a necessity to establish successful high-throughput pipelines [as argued before (Calapai et al., 2022)] and is still a significant challenge. A common approach is to employ tracking devices for animals, such as colored jackets, collars, and a combination of video monitoring or electronic devices such as RFID chips; to allow identification (Andrews and Rosenblum, 1994; Fagot and Bonté, 2010; Rose et al., 2012; Gazes et al., 2013; Ballesta et al., 2014; Maddali et al., 2014; Tulip et al., 2017; Calapai et al., 2022). Due to a combination of physiological and technical issues related to implanting and reading RFID chips in large animals such as macaque monkeys (Fagot and Bonté, 2010), we opted for a picture-based identification algorithm that employed a convolutional neuronal network (Witham, 2018; Butler and Kennerley, 2019; Schofield et al., 2019; Jacob et al., 2021). With our network, the classification accuracy for individual animals was in line with the reported accuracy achieved for rhesus macaques using similar methods (Witham, 2018; Butler and Kennerley, 2019) to allow individualized autonomous training. However, we found that running the recognition algorithm twice (at the beginning of each trial) only marginally improved the network performance compared to running the algorithm once per trial. With trials longer than a few seconds (in contrast to our experiment) this strategy could more significantly improve recognition accuracy. We finally suggest that taking a picture from multiple vantage points would improve recognition significantly. Overall, this technique revealed to be reliable in efficiency and easy to implement in a python-based task control. Nonetheless, we believe that further optimizations are needed to establish for example: (1) an unsupervised and automatic updating of the network as well as (2) an internal quality control system to evaluate tagging accuracy. Based on the rapid advancements in machine learning, this technique will continue to improve to be suited for non-invasive real-time animal classification in social groups.

### Insights into turn-taking and social dynamics

It is essential to note that due to the low number of animals and the low engagement of animal F in Group 2, the following analysis will focus mainly on Group 1, and it is intended to be taken as a proof of concept. We observed no fighting or substantial behavioral alteration in our animals throughout the experiment. All animals who interacted with the device across several sessions (5 out of 6) could do so by taking turns. In the early steps of our automated protocol, there were strong differences in the level of engagement across animals, presumably as a result of social dynamics present in a small captive group of primates. Previous reports have shown that the social rank of animals affects their access to resources (Barton and Whiten, 1993; Boogert et al., 2006), with lower-ranked individuals having the least access. Since a trainer (or training device) may be seen as a resource by the animals, engagement in training might be influenced by the social rank of the animals (Prescott and Buchanan-Smith, 2003). However, it has also been suggested that low-ranking individuals performed better at cognitive tasks than higher-ranked individuals when isolated from the rest of the members (Bunnell and Perkins, 1980; Drea and Wallen, 1999; Reader and Laland, 2001), indicating that a failure to learn a specific task in low ranked individuals might be a consequence of personality rather than social ranking (Wergård et al., 2016). While a comparison between individually trained animals when separated from their social group and our group-based training would have helped us to elucidate the difference in performance relative to the social context, we decided not to focus on such comparison as temporary social isolation could have negatively impacted the welfare of the animals. Finally, as a detailed ethological assessment of group hierarchy was not available for our groups, an in-depth comparison with previous studies is not possible. Our analysis revealed a specific non-random structure in the animal turn-taking that was stable across several months. This proof of concept represents an encouraging step forward toward the development of efficient and standardized techniques to assess NHPs’ social states and dynamics.

In summary, we described a study with 6 captive long-tailed macaques (across two groups) who were given access to a touchscreen device equipped with a step-wise automated training protocol and picture-based, real-time animal identification. Across 3 months of daily 3 h sessions (Monday to Friday, 10:00 to 13:00), animals successfully learned the basics of a visually guided discrimination task. Still, they failed to generalize to an acoustic-only discrimination task. Furthermore, in structured turns, animals interacted with the device in a self-paced manner, without fluid/food control nor social separation, with the likelihood of initiating a trial getting independent from the trial outcome as the performance drops to chance.

## Materials and methods

All animal procedures of this study were approved by the responsible regional government office [Niedersächsisches Landesamt für Verbraucherschutz und Lebensmittelsicherheit (LAVES), protocol number: 33.19-42,502-04-16/2278] and were in accordance with all applicable German and European regulations on husbandry procedures and conditions.

### Animals

Six female long-tailed macaque monkeys (*Macaca fascicularis*) housed in two groups were involved in this study (Group 1 with four animals: B, K, L, and R and Group 2 with two animals: G and F; see Table 1 for more details about the animals). The animals were group-housed in the facilities of the German Primate Center (DPZ) in Goettingen, Germany, equipped with an enriched environment including a multitude of toys and wooden structures, natural as well as artificial light and exceeding the size requirements of the European regulations, including access to outdoor space. The animals’ psychological and veterinary welfare was monitored by the DPZ’s staff veterinarians, the animal facility staff, and the lab’s scientists, all specialized on working with non-human primates. During the testing sessions, animals were fed their regular diet and water *ad libitum*. Training sessions took place mostly in the morning before the feeding time, with a single session taking place in the afternoon. The regular duration of a session was around 2 to 3 h, where the system was attached to the cage for animals to interact with at their own pace. Animal R (4 sessions) and F (2 sessions) were separated for individual training, while all remaining sessions were conducted with all animals having access to the device as a group.

### Apparatus

Data were collected with a custom-made, autonomous, touchscreen device tailored toward macaque monkeys (Calapai et al., 2017) and based on two python-based computers [Raspberry Pi; adapted from Calapai et al. (2022)]. The device was modified to deliver acoustic stimulation *via* two speakers located at the upper left and right corners of the device. The Long-tailed Experimental Behavioral Instrument, in short LXBI (50 × 57 × 30 cm − HxWxD) operates as an unsupervised, standalone, waterproof device that can be attached directly to the home enclosure of the animals *via* a custom-made frame (Figure 1). The device comprises two Raspberry Pi single-board I/O computers (Raspberry Pi 3B+, raspberry.org) to control the experiment and provide real-time video monitoring; a camera module attached to the task controller for animal identification (Raspberry Pi wide-angle camera module RB-Camera-WW Joy-IT); a capacitive touchscreen (15-inches touchscreen, ELO 1537 l SecureTouch); two peristaltic pumps (Verderflex OEM-Schlauchpumpe M025 DC, 10-30 V, 6,5 W) and a custom-made reward tube (placed at 25 cm distance from the screen); and two speakers (Visaton FR58, 8 Ω, 120–20,000 Hz). All components operated at low voltage—between 5 and 12 v—at a maximum of 2.5 A (touchscreen).

### Picture-based animal identification

During AUT experimental sessions, when an animal triggered the start stimulus, a picture was taken from the front camera (left panel of Figure 1A), downsampled to 300 × 300 pixel, converted to gray values, and fed into a custom-made, convolutional neural network optimized for object categorization (inspired by LeCun et al., 2015), to label the picture with one of the animals’ identities. A second picture was taken (in later sessions) to increase the robustness of the identification of a given animal. This second picture followed the same processing of the first picture described above.

### Structure of the network

The network was designed, trained, and used during the experiment through the *TensorFlow* module (Abadi et al., 2016), version 2.0; under Python 3.7. The network consisted of 9 layers in total, from input to output: an Average Pooling input layer (6 × 3 pooling size); 3 convolutional layers (3 × 3 kernel, ‘relu’ activation function, with 64, 16, 32 neurons, respectively); 3 pooling layers (MaxPool 2 × 2; Dropout; Flatten); 1 Dense layer (with a ‘relu’ activation function); and a final Dense output layer (with a ‘softmax’ activation function). The network was compiled with an ‘adam’ optimizer, a sparse categorical ‘crossentropy’ function, and ‘accuracy’ as metrics. The fitting was done in 10 epochs and with a batch size of 32. The output layer, representing the animals in each group, contained an additional neuron, here called *null*, that was trained on pictures triggered by the animals by accident (e.g., with their back). Parameters include the size of the average pooling kernel; the number of neurons in the three convolutional hidden layers; and the number of neurons in the hidden dense layer; they were all bootstrapped beforehand on the platform Google Colab.^1^ Here, with a test dataset of 3,000 pictures of two male macaque monkeys taken with the same device and in the same facility, we trained and tested 46 combinations of the parameters mentioned before. Finally, we compared the performances of the 46 resulting networks and handpicked the combination of parameters of the network with the highest accuracy (98.7%). This combination was used as the final configuration for the network used during the experiment.

### Training and maintenance of the network

The initial training set was collected in 2 weeks (10 sessions) during the experiment’s first phase and consisted of 300 pictures per animal. The network was retrained again after 5, 29, and 32 sessions for Group 1; and 4 sessions for Group 2, to account for possible changes in environmental factors from day to day. Every picture collected in both phases of the experiment was labeled by one of the experimenters, which was already very familiar with the animals, with a custom-made python interface. Labels were used to train and assess the network throughout the study.

### Procedure

The following training procedure is an adaptation of a protocol already described for common marmosets (Calapai et al., 2022). However, substantial changes regarding the dimensions and the identity of the stimulus were made. Therefore, the following description aims at highlighting the differences from the marmoset study. In order to run a session, a LXBI device was first attached to the animal’s cage and then turned on; leading to automatic starts of a local camera server for remote monitoring and video recording; the mount of a local network server for recursive data logging; and the loading of custom-made graphical user interface that allowed the experimenter to set up the parameters of the session (if needed) and launch the experiment. During this time, the reward (Pineapple, banana, or grape juice at 25% dilution with water) was loaded on the bottles of the reward system and manually pumped along the tubes that led to the mouthpiece (with a custom circuit operated by a momentary switch). Once the device was ready, the experimenter granted access to the device to the animals by removing a panel that divided the LXB from the group’s cage. The LXBI was left in the cage while remote surveillance took place every 15–20 min. At the end of the session, the panel was placed back, allowing the experimenter to open the device from one side (*via* dedicated hinges) and clean it thoroughly. Pumps were left to run for 30 min with hot water to clean debris, and if needed, the device was removed from the animal’s cage and stored for the next session.

### Sessions

Most of the sessions took place in the morning from 10:00 to 14:30 with two exceptions that extended until the afternoon (10:00 to 17:00). Food was provided at 14:00 by facilities’ caretakers, and water was available *ad libitum* throughout the session. For most sessions, videos of the animals working on the LXBI were recorded.

### Manual training sessions

Even though all animals had prior interaction with touchscreen devices, animals R and F underwent individual training sessions after we noticed that they did not adequately interact with the touchscreen. These animals were separated from the group for 4 (animal R) and 2 (animal F) sessions, during which, through PRT shaping techniques, they were manually trained to touch the screen to receive the reward.

### Experimental paradigm

Across and during all the sessions, animals never left their home cage. Except for animals R and F, which underwent 4 and 2 manual training sessions, the remaining 4 animals did not require manual training to operate the device. All animals underwent a series of picture-taking sessions (10 sessions) necessary to collect training pictures for the identification network. After this phase, all animals underwent an autonomous, unsupervised training protocol (AUT) comprised preconfigured training steps to acquire basic proficiency in a standard 2 AC discrimination task. It is essential to highlight that all sessions for both groups were gathered with a single LXBI device, and the data collection occurred in successive order, first for Group 1 followed by Group 2.

### Picture taking

A total of 10 sessions per group were designed to collect as many pictures from the animals as possible to train the picture-based identification algorithm. A simple behavioral task was built for this purpose. The animals were presented with a white screen, and every time a touch was registered, a picture was taken, labeled, and stored, while the reward pump delivered 1.5 ml of juice during the first session to attract the attention of the animals and then reduced to 0.5 ml. The animals had to wait for 2 to 3 s between one trial and the next.

### Automated unsupervised training

After the picture-taking sessions, all animals started an automated training procedure comprised of 50 steps. During the first 49 steps, an algorithm would gradually guide the animals according to their performance (Berger et al., 2018), while in step 50 no change in the task occurred (Calapai et al., 2022). In the AUT section (steps 1–49), animals had to learn the basic rules of a visuo-acoustic 2 AC, in which the proficiency of an animal was assessed at step 50. Specific parameters, such as size, location, and time delays, were adjusted during the AUT. Animals could step up when 8 or more out of 10 trials were correct and step down when 2 or less out of 10 were correct. During and across sessions, the progress of every animal was stored and retrieved every time an animal started to perform a trial. In this way, animals could individually navigate the total series of steps resuming after pauses or end of sessions at the same step they left in the last interaction. As mentioned above, the present training protocol is an adaptation from an AUT developed for marmosets (Calapai et al., 2022), with the main differences found in the stimuli’s identity and stimuli configuration. The AUT was comprised 49 steps, grouped into 3 milestones that aim at training long-tailed macaques on the basics of touchscreen interactions within the context of a visuo-acoustic 2 AC task. (1) *Size* milestone (steps 2–15) aimed to train animals to execute precise touches by decreasing the size of the visual stimulus that triggered the reward. A white circle embedded in a blue rectangle (called *trigger*) placed in the center of the screen had to be touched to obtain the reward (0.5 ml). Throughout the steps, the trigger gradually decreased from 20 cm × 20 cm to 6 cm × 6 cm. Touching outside the trigger resulted in a 5–7 s long inter-trial interval signaled by a gray screen, during which screen touches were ignored and resulted in a reset of the inter-trial interval. In contrast, touching inside the trigger would allow a new trial initiation after a randomized inter-trial interval of 2–4 s. (2) *Location-sound* milestone (steps 16–30). Here the AUT attempted to train animals to associate a sound with a visual target while also improving the spatial precision touch behavior. Throughout the steps, the trigger appeared at the center of the screen, and upon touch, an acoustic stimulus (either a repeated infant long-tailed macaque vocalization; or a pure tone train of 4 kHz at variable loudness – see below) was presented for 1–1.5  s before a visual target appeared, at step 16 (the first of this milestone) the visual target appeared in the center of the screen, but gradually moved away, to either side of the screen, by 1 cm on each step until reaching the edge. In contrast, the side randomly changed from left to right on a trial-by-trial basis. The visual targets consisted of a picture of an infant long-tailed macaque face (6.5 × 6.5 cm), or an abstract geometric pattern (6.5 cm × 6.5  cm; Figure 2A). The vocalization was matched with the long-tailed macaque face while the pure tone trained with the geometric pattern. Along the steps of the *location-sound* milestone, the intensity of the sound was gradually increasing (in steps of 10 dB) from 30 ± 2 dB SPL on step 16 to a final loudness of 72 ± 2 dB SPL on step 22. (3) *Distractor* milestone (steps 31–49). Here, the AUT trained the animals to discriminate the two visual targets by introducing a second visual target as a distractor with a smaller size than the target. A second visual target (distractor) was displayed together with the first target but on the opposite side of the screen, at the same eccentricity. In the case of a ‘vocalization’ trial, the visual distractor was the geometric pattern and vice versa. The distractor was gradually increased in size on each step from 0.3  cm × 0.3  cm in step 31 up to 6.5  cm × 6.5 cm in step 50 (step:size – 31:0.9 cm^2^, 32:1.8 cm^2^, 33: 2.56 cm^2^, 34: 4.84 cm^2^, 35: 8.41 cm^2^, 36: 11.55 cm^2^, 37: 13.69 cm^2^, 38: 15.21 cm^2^, 39: 17.64 cm^2^, 40: 20.25 cm^2^, 41: 22.09 cm^2^, 42: 25 cm^2^, 43: 28.09 cm^2^, 44: 32.49 cm^2^, 45: 34.81 cm^2^, 46: 36 cm^2^, 47: 38.44 cm^2^, 48: 39.69 cm^2^, 49: 40.96 cm^2,^ 50:42.25 cm^2^) at which point it reached the same size as the target. Throughout the protocol, the lack of further interaction within 8 s after trial initiation resulted in an aborted trial, and the trial outcome was labeled as ‘ignored.’ The AUT aimed to instruct the animals in a visuo-acoustic discrimination experiment. They had to distinguish two different sounds and select the corresponding visual stimulus to indicate their choice.

### Acoustic-only discrimination task

After having completed the AUT protocol, and therefore having reached step 50, animals were presented with an acoustic-only 2 AC task in which they had to discriminate a vocalization from a pure tone train and report their choice by touching the correspondent visual target on the screen. As mentioned earlier, the vocalization was associated with the picture of an infant long-tailed macaque, whereas the pure tone train with a geometric pattern. This association was instructed during the AUT protocol (steps 1–49). In contrast to the AUT protocol, in step 50, animals were required to rely solely on acoustic cues to identify the target of a given trial and thus obtain the reward above chance. A trial was counted as correct when an animal could respond to the sound with the correct visual target on the screen and rewarded with 0.5 ml of juice, followed by a 1–2 s timeout. When the animal chose the wrong visual target, the screen turned gray for 8 s, during which interactions with the touchscreen were ignored. Throughout this task, the lack of further interaction within 8 s after trial initiation resulted in an aborted trial, and the trial outcome was labeled as ‘ignored.’

### Data treatment and statistics

Data acquisition, formatting, and analysis were performed using Python 3.5.3 and 3.7.7. All figures, means, and medians were calculated using the Python libraries *Numpy*, *Pandas*, *Seaborn*, and *Matplotlib*. Double-sided Pearson’s correlations were calculated using the module *pingouin*. Psychometric functions for obtaining thresholds in size difference were calculated using the module *psignifit* (Schütt et al., 2016) and setting the fit to cumulative normal sigmoid function, with all parameters free and with 95% confidence intervals. This resulted in the following function:


$$\psi \left(x;m,w,\;\lambda ,\;\gamma \right)=\gamma +\left(1-\lambda -\gamma \right)S\left(x;m,w\right)$$

Where *m* represents the threshold (the level at 0.5), *w* represents the width (difference between levels 0.5 and 0.95), *λ* and *γ* represent the lower and upper asymptote, respectively.

## Data availability statement

The raw data supporting the conclusions of this article will be made available by the authors, without undue reservation.

## Ethics statement

The animal study was reviewed and approved by Niedersächsisches Landesamt für Verbraucherschutz und Lebensmittelsicherheit (LAVES).

## Author contributions

AC and MJ conceived the study. JCM, AC, and MJ designed the experiment and interpreted the data. LJ performed the experiments. JCM analyzed the data and wrote the manuscript with input from MJ and AC. All authors contributed to the article and approved the submitted version.

## Funding

This work was partially funded by an Audacity grant (LSC-AF2020_05) of the Leibniz Science Campus’ Primate Cognition’ to AC and MJ.

## Conflict of interest

The authors declare that the research was conducted in the absence of any commercial or financial relationships that could be construed as a potential conflict of interest.

## Publisher’s note

All claims expressed in this article are solely those of the authors and do not necessarily represent those of their affiliated organizations, or those of the publisher, the editors and the reviewers. Any product that may be evaluated in this article, or claim that may be made by its manufacturer, is not guaranteed or endorsed by the publisher.

## Footnotes

^1^ https://colab.research.google.com/
